# Intraoral Neurinoma of the Lingual Nerve: An Uncommon Tumor in Floor of the Mouth

**DOI:** 10.1155/2014/385068

**Published:** 2014-01-30

**Authors:** Santhosh Kumar kuppusamy, Subramaniyam Ramkumar, Malathi Narasimhan, Emmanuel Azariah Dhiravia Sargunam

**Affiliations:** Department of Oral & Maxillofacial Pathology, Faculty of Dental Sciences, Sri Ramachandra University, Porur, Chennai, Tamil Nadu 600116, India

## Abstract

Neurinoma or schwannoma is an uncommon benign tumor that arises primarily from the nerve sheath of Schwann cells. About 25% has been reported in head and neck region extracranially, but only 1% in the intraoral origin. Intraorally, the tongue is the most common site followed by the palate, floor of the mouth, lips and buccal mucosa. In review of literature, intraoral schwannoma of the lingual nerve origin has not been reported frequently. So, we present a case of intraoral neurinoma of the lingual nerve.

## 1. Introduction

Schwannoma/neurinoma is a nerve sheath tumor that originates from the Schwann cells of peripheral, cranial, and autonomous nerves. It is a slow growing benign tumor, mostly asymptomatic in nature. It frequently appears as a solitary encapsulated swelling, except the neurofibromatosis type which occurs as multiple lesions [1]. Malignant transformation of the lesion is extremely rare, making complete surgical excision to be the treatment of choice [2]. Incidence of neurinoma in the intraoral region is about only 1%, in which tongue is the common site followed by palate, floor of the mouth, lips, and buccal mucosa [1–4].

In the literature, there are reported cases of neurinoma/schwannoma originating from the mylohyoid nerve, hypoglossal nerve, and sublingual gland, but there are a very few literatures on schwannoma originating from the lingual nerve [1]. Subhashraj et al. reviewed the English medical literature and concluded that, including their case, till now only eight cases of ancient schwannoma have been reported with a maximum diameter of 31 mm in males and 55 mm in female patients [4]. Here we present a very rare case of neurinoma from the lingual nerve with a rapid growth in a relatively shorter duration and a review of the literature.

## 2. Case Report

**Figure 1:**
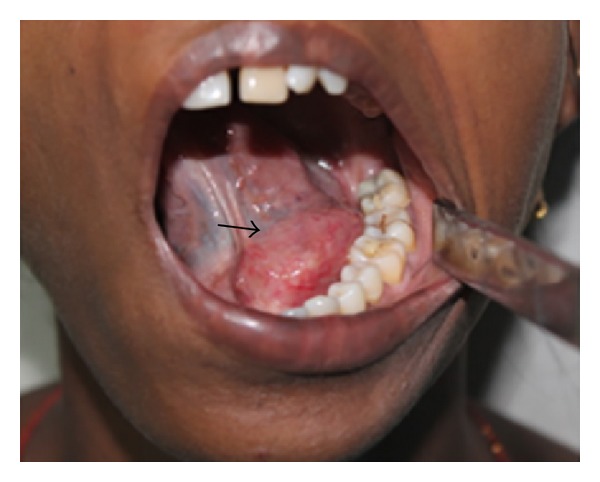
Intraoral view of well-circumscribed swelling (black arrow) in the left floor of the mouth.

A thirty-one-year old female patient reported to the Department of Oral and Maxillofacial Surgery with a swelling in the floor of the mouth for the past two months. The swelling was initially small and gradually reached the present size over two months. On extraoral examination, a diffuse swelling in the left submandibular region was present. On intraoral examination, a single well-defined encapsulated swelling was present in the left side of floor of the mouth (Figure 1). On palpation, the swelling was tender, firm, and nonfluctuant and the swelling extends anteroposteriorly from the midline of the floor of the mouth to the second molar region in the left side of the mandible (Figure 1). Patient was having altered sensations in the left lateral border of tongue and the lingual mucoperiosteum of the left side of mandible which suggested that the mass related to the lingual nerve. FNAC was found negative as the swelling was firm.

**Figure 2:**
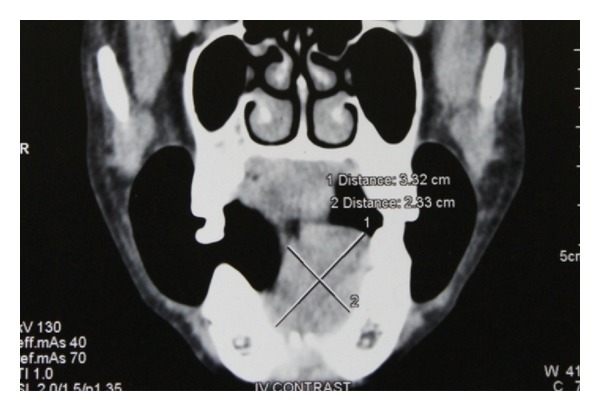
Axial CT shows dense mass of 3.32 cmm 2.33 cmm in the floor of the mouth.

**Figure 3:**
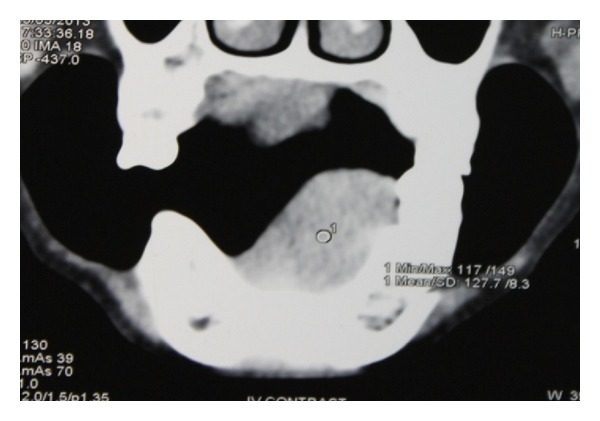
Axial CT shows lingual nerve (small circle) found within the mass in the left floor of the mouth.

Axial computed tomogram of head and neck showed a dense mass in the left side of floor of the mouth region measuring about 33 mm × 23 mm with a dilated lingual nerve found within the mass (Figures 2 and 3).

**Figure 4:**
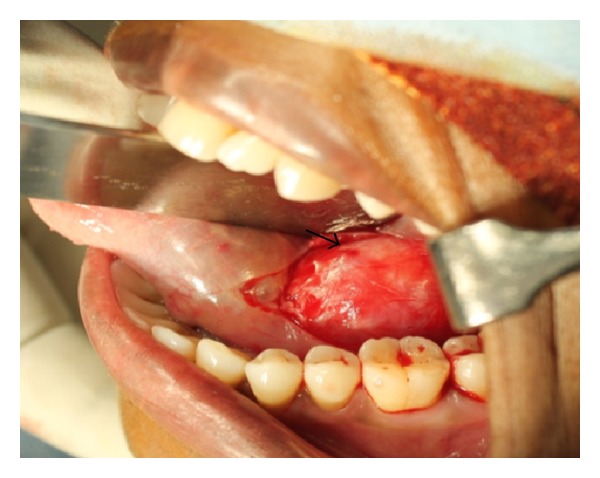
Intraoperative picture showing mucosal incision and exposed encapsulated mass (black arrow) in the left floor of the mouth.

**Figure 5:**
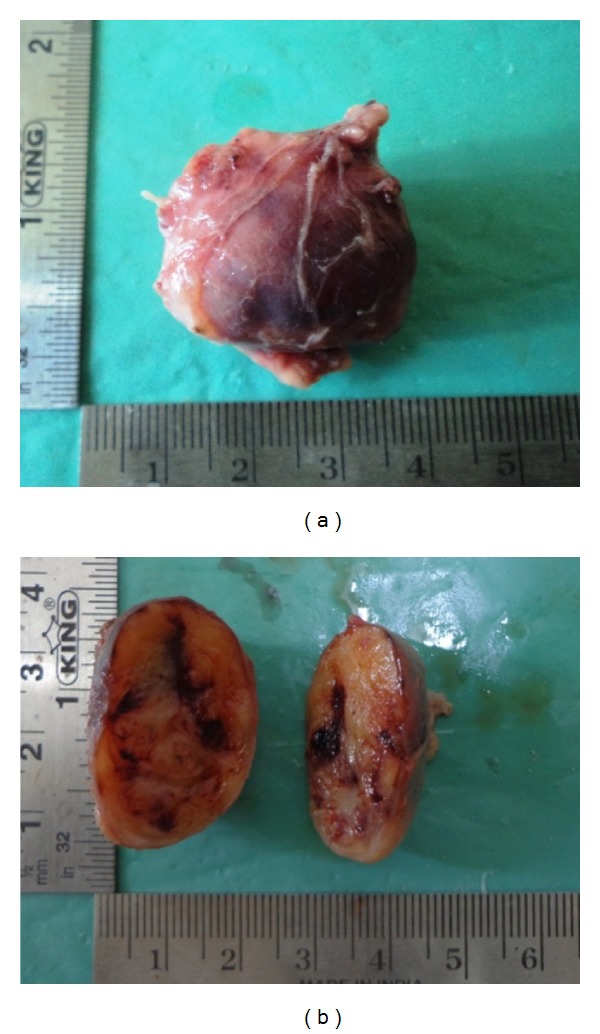
(a) Macroscopic picture of well-encapsulated mass measuring 3.5 cm-4 cm-3 cm. (b) Cut surface of specimen shows areas of cystic degeneration inside surrounded by solid area.

**Figure 6:**
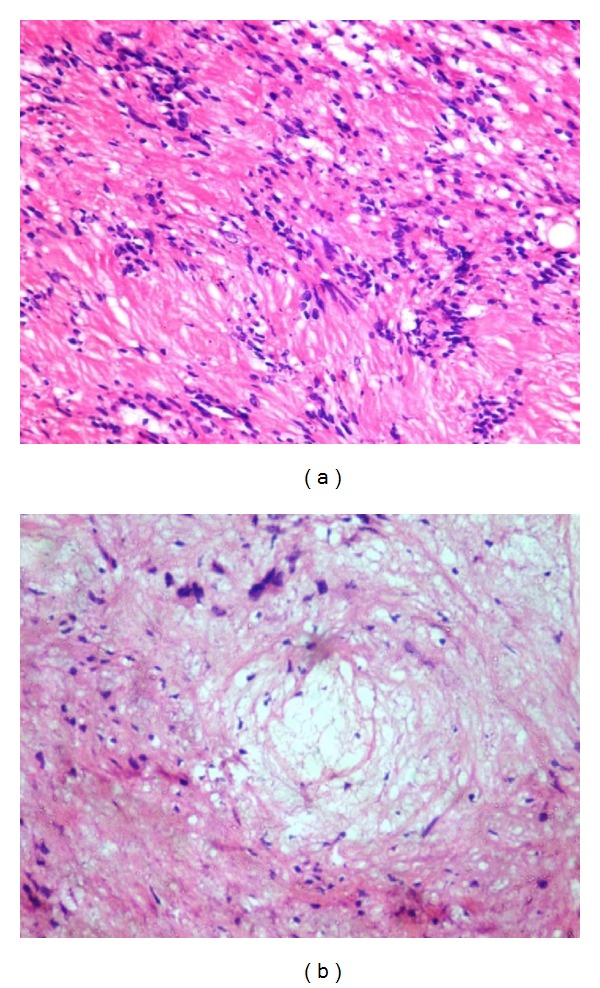
(a) Histopathological view of 10x shows Antoni A: cellular region, nuclei palisaded in arrangement around central acellular eosinophilic areas, and Verocay bodies. (b) Histopathological view shows Antoni B: relatively acellular in a loose, myxomatous stroma.

Under general anaesthesia, a mucosal incision was placed over the mass in the left side of the floor of the mouth, the tumor was exposed and dissected safely from the lingual nerve, and excision was carried out by ligating the lingual nerve on the distal and mesial end of the swelling (Figure 4). The encapsulated tumor was then excised completely from the lingual nerve and the lingual nerve repair was done by perineural suturing. Macroscopic examination of the excised specimen reveals a well-encapsulated mass and the cut surface of the specimen measures about 3.5 cm × 4 cm × 3 cm showing an area of cystic degeneration and solid areas (Figures 5(a) and 5(b)). Histopathological examination showed fibrous capsule enclosing proliferation of Schwann cells with characteristic Antoni A and Antoni B types of tissue with areas of cystic and myxoid degeneration, suggestive of schwannoma (Figures 6(a) and 6(b)). Patient was then followed up postoperatively for six months without any recurrence and complications.

## 3. Discussion

The term “ancient schwannoma” was coined by Ackerman and Taylor as it shows long standing degenerative changes in the benign neural tissues and has a distinctive area of hypocellular tissues [2, 4, 5]. Clinically, intraoral neurinoma occurs as two types—either an encapsulated or nonencapsulated lesion. But histologically, it exhibits five variants—common, plexiform, cellular, epithelioid, and ancient schwannomas [2, 4].

According to English the literature review, to date a total of only eight cases of ancient schwannoma have been reported. Nakayama et al. reported a maximum diameter of lesion in female patient of 55 mm; Dayan et al. reported ancient schwannoma with a maximum diameter of 31 mm [4, 6, 7]. Interestingly in this review, apart from the duration and the size of the lesion, there is a lack of clinical data related to pain and neurological disturbances in ancient schwannoma. Axial CT section of our patient showed a schwannoma of 33 mm with a dilated lingual nerve found within the mass. Relatively our patient has pain in the left floor of the mouth over the swelling during swallowing and neurological disturbances in the floor of the mouth and lingual mucoperiosteum which is uncommon in schwannoma of intraoral origin.

Differential diagnosis of the swelling in the floor of the mouth can be a mucocele, ranula, pleomorphic adenoma, or Submandibular gland enlargement. Most of the cases in the floor of the mouth, FNAC is inconclusive as it always gives negative result either when swelling is firm or it gives false results mostly as pleomorphic adenoma of submandibular gland in cases of intraoral schwannoma of submandibular gland [7].

The clinical and radiological diagnosis will not give reliable results like a histopathological diagnosis. Complete surgical excision and histopathological examination of the excised lesion could be a choice for treatment of an encapsulated mass in the floor of the mouth [2]. Similarly we performed the complete surgical excision with neural repair of lingual nerve on the left side of floor of the mouth. During the postoperative period, the patient showed a normal neurosensory function in a two-week period.

Ancient schwannomas possess four unique features: (1) encapsulated mass with degenerative alterations containing both large cystic and solid areas, (2) mixture of spindle cells with highly cellular (Antoni A) and less cellular myxoid (Antoni B), (3) palisaded nuclear appearance of Schwann cells, (4) and Verocay bodies seen in a cellular eosinophilic zone. Our case is also an ancient schwannoma because it has all the unique features such as the encapsulated mass in the floor of the mouth and excised gross specimen examination shows both large cystic and small solid areas in it; similarly histopathological features show fibrous capsule enclosing proliferation of Schwann cells with Antoni A and Antoni B types of tissue which suggest ancient schwannoma [2, 8].

Significance of our case is the rapid growth with a maximum diameter of 4 cm, associated with pain and neurosensory disturbance in a shorter duration of two months which is not a unique character of intraoral neurinoma, and this makes our case different from the previously reported cases.

## 4. Conclusion

Intraoral neurinoma is not a frequently encountered benign neoplasm and histopathological examination is the only reliable method for diagnosis of submucosal lesion. Malignant transformation of intraoral neurinoma has not been reported yet, so complete surgical excision has a good prognosis for the treatment of intraoral neurinoma. In spite of lack of the literature on the clinical significance of neurosensory disturbances in intraoral neurinoma, we advise that preservation of nerve during complete surgical excision and microsurgical repair of nerve ending play a vital role in recovery and prevention of neurosensory disturbances in the postoperative period.
